# Common Ragweed Allergy Under Global Change Linking Invasion-Driven Aeroallergen Exposure with Molecular Sensitization and Allergic Airway Disease

**DOI:** 10.3390/life16091431

**Published:** 2026-08-28

**Authors:** Maria Alexandra Ferencz-Iepan, Lavinia Ștef, Sandra Florina Lele, Florica Emilia Morariu, Nicolae Corcionivoschi, David McCleery, Igori Balta, Ioan Peț

**Affiliations:** 1 Faculty of Bioengineering of Animal Resources, University of Life Sciences King Mihai I from Timisoara, 300645 Timisoara, Romania; maria.ferencz@usvt.ro (M.A.F.-I.); laviniastef@usvt.ro (L.Ș.); sandra.lele@usvt.ro (S.F.L.); floricamorariu@usvt.ro (F.E.M.); nicolae.corcionivoschi@afbini.gov.uk (N.C.); david.mccleery@afbini.gov.uk (D.M.); ioanpet@usvt.ro (I.P.); 2 Doctoral School “Engineering of Vegetable and Animal Resources”, University of Life Sciences “King Mihai I” from Timişoara, Calea Aradului 119, 300645 Timisoara, Romania; 3 Academy of Romanian Scientists, Ilfov Street 3, 050094 Bucharest, Romania; 4 Bacteriology Branch, Veterinary Sciences Division, Agri-Food and Biosciences Institute, Belfast BT4 3SD, Northern Ireland, UK

**Keywords:** common ragweed (*Ambrosia artemisiifolia*), biological invasion, aeroallergen-bearing particles, pollen allergenicity, climate change, component-resolved sensitisation, airway inflammation, molecular allergology

## Abstract

The case of common ragweed (*Ambrosia artemisiifolia*) stands as a prime example of an invasive plant that links global change, biological invasion, aeroallergen exposure, and allergic airway disease. However, evidence remains fragmented across invasion biology, aerobiology, molecular allergology, and respiratory medicine. By separating plant occurrence, pollen abundance, molecular allergen dose, sensitisation, and airway disease, this review develops an integrated invasion–exposure–disease logic for interpreting ragweed-related health risk. We examine how climate change, land-use disturbance, repeated introductions, rapid adaptation, and air pollution influence plant distribution, flowering phenology, pollen production, airborne allergen load, and respiratory outcomes. Attention is given to Amb a 1 as the principal marker of genuine ragweed sensitisation, cross-reactivity with *Artemisia* and other weed pollens, allergen-bearing respirable particles, and the diagnostic limitations of extract-based immunoglobulin E (IgE) testing. The literature indicates that ragweed sensitisation follows a pronounced hotspot–gradient pattern in Europe, whereas patterns in other invaded regions remain more heterogeneous and incompletely characterised. Clinically relevant exposure depends not only on pollen concentration but also on airborne allergen load, pollen allergen potency, atmospheric transport, respirable particle fractions, meteorological conditions, and pollution. Ragweed-related airway disease is mediated by IgE-dependent type 2 immunity and amplified by epithelial danger signals, oxidative stress, protease activity, and innate immune pathways. Based on current evidence, we propose that an integrated surveillance framework linking plant distribution, pollen and airborne-allergen exposure, molecular sensitisation, symptoms, lung function, and asthma outcomes could strengthen risk forecasting, source attribution, prevention, and invasion control of the common ragweed.

## 1. Introduction

*Ambrosia artemisiifolia* L. (common ragweed) is now among the most significant invasive alien plant species at the intersection of weed science, aerobiology, and public health. Although the genus includes multiple species, it is the dominant invasive ragweed in Europe and a leading global contributor to pollen-related morbidity, owing to its exceptional ecological flexibility, high reproductive capacity, and effective colonisation of disturbed habitats [1,2,3]. Native to North America, it was introduced to Europe in the nineteenth century mainly through contaminated seed and trade materials, subsequently spreading along transport and disturbance networks such as roadsides, railways, waterways, and agricultural machinery across central and eastern Europe, including major hotspot regions [4,5,6]. Historical herbarium records suggest that France served as an early entry point for the species’ spread in Europe, after which it rapidly expanded across Central and Eastern Europe, including Hungary, Ukraine, Russia, and Romania [5].

The invasive success of *A. artemisiifolia* is supported by biological traits that promote persistence and rapid expansion across landscapes. The species is highly fertile, often producing several thousand seeds per plant, with reported maximum outputs far exceeding this under ideal conditions, and its seed bank can stay viable in soil for decades [3]. It establishes especially well in ruderal environments such as disturbed, open, and transport-related habitats, where it quickly develops and spreads [7]. In agricultural fields, it competes fiercely with crops for light and nutrients, releases phytotoxic and allelopathic compounds, and causes measurable agricultural losses in cereals, soybeans, sunflowers, and other production systems. This makes *A. artemisiifolia* relevant not only as a weed but also as an agronomic concern [3,8,9].

From a medical perspective, its main impact is mediated by pollen emissions. In heavily affected regions, high airborne pollen concentrations are associated with seasonal allergic rhinitis/rhino-conjunctivitis and clinically relevant lower-airway involvement, including asthma symptoms and exacerbations in sensitised individuals [10]. A recent systematic analysis from the Global Burden of Disease study estimated that asthma affected approximately 260 million individuals worldwide in 2021, underlining the scale of chronic respiratory vulnerability in which seasonal triggers operate [11]. Critically, the clinically relevant dose is shaped not only by source strength but also by meteorology, pollution, and atmospheric processing that influence allergen delivery-including conditions that favor rupture and the production of respirable allergen-bearing fractions relevant to acute exacerbations, such as thunderstorm-asthma phenomena, which—although most extensively documented for grass pollen [12,13] and fungal spores [14]—have also been linked mechanistically to other allergenic pollen types, including olive [15] and Asteraceae family weeds [16]. Importantly, airborne pollen monitoring provides the essential baseline for assessing exposure to *A. artemisiifolia*. Still, health risk is also shaped by meteorological conditions, air pollution, atmospheric transport, and the biological presentation of allergenic material, including identifiable pollen fragments and respirable allergen-bearing particles. Pollutants may adhere to pollen grains, forming pollen-particle complexes and potentially amplifying allergenic effects. At the same time, high humidity, rainfall, and thunderstorm conditions can promote pollen rupture and the formation of fine, respirable allergen-bearing particles associated with acute respiratory exacerbations, including thunderstorm-related asthma [17,18]. In addition, *A. artemisiifolia* pollen may co-occur with allergenic fungal spores (e.g., *Alternaria*, *Cladosporium*, *Aspergillus*, *Penicillium*) and yeasts, further complicating exposure mixtures and potentially intensifying symptom severity in susceptible individuals [17].

Overall, the available evidence identifies *A. artemisiifolia* as a key invasive species whose importance goes beyond weed control. It is simultaneously an ecological invader, an agricultural stressor, an atmospheric bioaerosol source, and a contributor to the burdens of allergic and asthmatic diseases, especially in areas where climatic suitability, human activity, and sensitised populations increasingly intersect [1,7].

Therefore, this study was designed as a structured integrative narrative review of *A. artemisiifolia* as a coupled invasion–aeroallergen–health system. It links climate and land-use drivers of spread with eco-evolutionary and genomic determinants of phenology and pollen output. It also examines the atmospheric processes that shape aeroallergen dose and the clinical pathways leading to sensitisation and symptom burden. This review aims to synthesise evidence on *A. artemisiifolia* as an integrated invasion–aeroallergen–health system under global change. Specifically, it examines how climate warming, land-use disturbance, biological invasion, eco-evolutionary adaptation, atmospheric pollen and allergen dynamics, and host immune responses interact to shape ragweed-related sensitisation, allergic rhinitis, and asthma risk. Operationally, this review integrates evidence from weed science, invasion genomics, aerobiology, environmental exposure assessment, molecular allergology, and respiratory medicine. To avoid conflating ecological, aerobiological, and clinical findings, these domains are first examined separately and then unified within an invasion–exposure–disease framework. The review therefore prioritises mechanistic pathways linking ragweed spread and pollen production to clinically relevant aeroallergen exposure and respiratory disease, rather than attempting an exhaustive account of the species’ biology and ecology.

## 2. Review Methodology

This study was conducted as a structured integrative narrative review rather than as a systematic review or meta-analysis. This approach was selected because the review question spans multiple disciplines and incorporates heterogeneous evidence, including ecological field studies, plant-growth experiments, population-genomic and transcriptomic investigations, aerobiological monitoring, atmospheric-transport modelling, clinical and pre-clinical studies, molecular-allergen research, and controlled allergen-challenge studies. The review addressed the following overarching question: how do invasion dynamics, environmental change, evolutionary adaptation, pollen and allergen exposure, molecular sensitisation, and airway immune mechanisms interact to determine the respiratory-health burden associated with *A. artemisiifolia*? The scope was intentionally interdisciplinary but was restricted to evidence contributing directly to at least one component of the proposed invasion–exposure–disease pathway.

Targeted literature searches were conducted in PubMed, Scopus, Web of Science Core Collection, and Google Scholar. The searches covered the period from database inception to June 2026. No lower publication-date restriction was applied because several older studies provide foundational evidence concerning ragweed allergen nomenclature, sensitisation, controlled airway challenge, pollen-associated oxidative activity, and invasion history. Nevertheless, we emphasised studies published during the preceding decade, particularly when recent evidence superseded or substantially refined earlier findings.

The core species-related search terms were “*Ambrosia artemisiifolia*”, “common ragweed”, “short ragweed”, and “ragweed”. These were combined with terms belonging to six thematic search blocks such as the following:

1. Invasion and distribution: “biological invasion”, distribution, establishment, propagule pressure, dispersal, disturbance, seed bank, range expansion, and management;
2. Adaptation and plant biology: genomics, transcriptomics, structural variation, haploblock, copy-number variation, adaptation, flowering time, phenology, growth, drought, temperature, and carbon dioxide;
3. Aerobiology and atmospheric exposure: pollen, aerobiology, aeroallergen, long-distance transport, pollen season, allergen load, allergen potency, particulate matter, pollution, meteorology, and climate change;
4. Molecular allergens and sensitisation: “Amb a 1”, “Amb a 11”, allergen, immunoglobulin E, sensitisation, component-resolved diagnosis, cross-reactivity, profilin, polcalcin, and cross-reactive carbohydrate determinants;
5. Clinical disease and airway mechanisms: allergic rhinitis, rhino-conjunctivitis, asthma, airway inflammation, epithelium, type 2 immunity, cytokines, eosinophils, mast cells, basophils, innate immunity, oxidative stress, and protease;
6. Surveillance and intervention: pollen monitoring, exposure forecasting, invasion control, allergen immunotherapy, biologic therapy, prevention, and public health.

Eligible sources included peer-reviewed full-length original research articles, systematic reviews, meta-analyses, high-quality narrative or integrative reviews, and authoritative standards or institutional reports directly relevant to the review framework. Human observational and clinical studies, controlled allergen-challenge studies, in vitro and ex vivo investigations, animal models, plant-growth experiments, genomic and transcriptomic studies, atmospheric-monitoring studies, transport and distribution models, and invasion-ecology studies were eligible because each addressed a distinct component of the invasion–exposure–disease pathway.

Evidence was synthesised qualitatively within the invasion–exposure–disease framework rather than pooled statistically. Studies were organised according to the component of the pathway they informed: invasion and establishment, adaptive plant traits, atmospheric exposure, molecular sensitisation, or respiratory outcomes. Human observational, clinical, and controlled-challenge studies were given greatest weight when drawing clinical inferences, whereas plant-growth, genomic, in vitro, animal, and modelling studies were used primarily to establish source dynamics, mechanistic plausibility, or experimentally supported pathways. Sources that did not contribute directly to one of the predefined thematic domains, and evidence from non-*A. artemisiifolia* systems without a necessary comparative or mechanistic role were not retained for synthesis. Owing to substantial heterogeneity in study designs, exposures, populations, and outcome measures, quantitative pooling was not undertaken.

Studies concerning other *Ambrosia* spp., unrelated pollen taxa, or broader allergic-airway mechanisms were included only when they provided necessary comparative or mechanistic context that could not be obtained directly from *A. artemisiifolia* studies. Such evidence was explicitly identified as indirect or supportive and was not presented as ragweed-specific evidence.

## 3. Historical Invasion, Current Distribution, and Projected Range Expansion of Ragweed

*A. artemisiifolia* is native to North America and has become a widely established invasive alien plant in several regions of Europe, Asia, and Australia. The expansion follows the historical transcontinental spread of *A. artemisiifolia* from its native North American range into Europe, Asia, and Australia. In Australia, the species should not be framed as a recent continent-wide invader, but rather as a historically established and geographically restricted invaded-range example. It has been recorded in Australia since the early twentieth century and remains mainly associated with eastern Australia, particularly New South Wales and Queensland, with more limited records from Victoria [19,20]. Across invaded regions, the species is strongly associated with disturbed or weakly competitive habitats, including degraded or overgrazed pastures, roadsides, waste areas, floodplains, riparian corridors, agricultural margins, and other open habitats created or maintained by human activity.

The present global distribution of *A. artemisiifolia* is uneven and historically contingent rather than uniform. In Europe, the most established invaded corridors occur in Central and Eastern Europe, including the Pannonian Plain, parts of the Balkans, northern Italy, the Rhône Valley, and other disturbed lowland or river-valley systems [5,6,21,22]. These regions combine suitable climatic conditions, repeated propagule input, open disturbed habitats, and agricultural or transport corridors that favour establishment and recurrent spread. By contrast, Atlantic and Nordic regions generally show weaker establishment histories and more fragmented occurrence, although continued introductions and climate change may alter this pattern [6,21,23].

Outside Europe, invasion patterns are more heterogeneous. China represents an important example of Asian spread: since its first detection in northeastern China in 1935, *A. artemisiifolia* has been reported in more than 20 provinces [24]. However, Asian distribution should not be treated as a single uniform pattern, because establishment depends on regional climate, geographical aspect, propagule pressure, land use, disturbance frequency, and populations’ capacity to complete reproduction. Similarly, the Australian case illustrates that long-term presence does not necessarily imply broad continental spread, because suitable disturbed habitats and repeated propagule pressure remain essential for persistence and expansion [19,20]. Future range expansion is expected to be shaped by the interaction between climate suitability and invasion processes. In Europe, the distribution of *A. artemisiifolia* is projected to shift northwestward under combined climate forcing and ongoing spread, particularly into regions with currently limited establishment, including parts of the UK and Scandinavia [6,25,26]. Global warming may relax temperature or phenological constraints in marginal regions, allowing more frequent completion of flowering and seed maturation.

Taken together, the current distribution of *A. artemisiifolia* remains strongly region-dependent. Europe contains the most extensive invaded hotspots outside North America, whereas invasion tendencies in Asia and Australia are more heterogeneous. Future expansion will depend not simply on increasing climatic suitability, but on whether suitable conditions coincide with successful establishment and reproductive persistence. Establishment reflects the interaction between climatic suitability, propagule arrival, and habitat disturbance. Human activity couples these processes by transporting seeds through contaminated seed, soil, machinery, and infrastructure networks while simultaneously generating open microsites in which recruitment is favoured [7,27,28,29,30]. This coupling explains the corridor-like expansion observed along agricultural and transport networks. For allergy risk, however, the relevant consequence is not disturbance itself but the establishment of persistent reproductive populations that can act as seasonal pollen sources.

Once established, high fecundity and persistent seed banks can maintain local source populations after incomplete control, while long-distance pollen transport can extend exposure beyond the plant’s immediate distribution [5,31,32]. Invasion history therefore defines the geography and persistence of pollen sources; the next section considers how rapid adaptation can modify when and how strongly those sources contribute to seasonal pollen exposure.

## 4. Rapid Adaptation of Ragweed Traits Relevant to Aeroallergen Exposure

For allergy risk, the relevant consequence of rapid adaptation is whether invaded *A. artemisiifolia* populations alter the timing or magnitude of pollen production. Repeated introductions and admixture maintain genetic variation on which local selection can act, whereas environmental conditions modify reproductive development and plant size. The health-relevant developmental transition is therefore flowering: temperature can alter reproductive development and pollen-associated traits, photoperiod contributes to latitudinal differentiation in flowering time, and elevated atmospheric carbon dioxide (CO_2_) can increase reproductive allocation, including male flower production [33,34,35,36,37]. These processes determine when established populations become active pollen sources and contribute to their potential seasonal source strength.

Successful establishment matters to aeroallergen exposure only insofar as it determines whether introduced plants survive to reproductive maturity. Germination and early growth in *A. artemisiifolia* are responsive to temperature and water availability and vary among populations, while persistent seed banks enable recruitment after disturbance or incomplete control [32,33,36,37]. Once established, environmental conditions influence plant size and reproductive development, thereby affecting the probability, timing and magnitude of subsequent pollen production [34,38,39,40].

The health-relevant developmental transition is reproductive maturation. Male development progresses from inflorescence initiation to anthesis and pollen release, so changes in flowering onset and duration directly alter the temporal window of pollen-source activity [41,42,43]. For the invasion–exposure pathway, these effects matter because they determine whether established populations achieve the phenology and reproductive output needed to generate substantial seasonal pollen exposure. Against this developmental background, repeated introductions and propagule pipelines create the demographic and genetic conditions for rapid adaptation.

### 4.1. Repeated Introductions Maintain Adaptive Variation

Multiple introductions from the North American native range have generated admixed invasive populations and maintained substantial genetic variation following establishment [5,19]. This standing variation provides the substrate for rapid local adaptation, including shifts in flowering time and plant size [40,44]. Multi-habitat experiments showed that seed inputs required for establishment vary by environment, with lower thresholds in disturbed margins and significantly higher requirements in closed or highly competitive habitats, consistent with the role of disturbance in reducing biotic resistance [45]. Establishment success is further influenced by propagule arrangement, with aggregated seed delivery outperforming evenly spaced sowing, a pattern that reflects real-world delivery via localized packets of contaminated goods, machinery, or soil movement [45]. In other words, ragweed seems to face its most significant low-density demographic constraints during initial establishment, whereas populations that successfully colonise a site become much less susceptible during subsequent early expansion.

A key ecological consequence of *A. artemisiifolia* invasion is the coupling of high fecundity, persistent seed banks, and long-distance pollen dispersal. In core invaded regions, plants typically produce around 1500–3000 seeds per individual, although much higher maxima have been reported under favourable conditions. Fecundity scales with vegetative biomass and early emergence [5,31,32]. In parallel, long-distance atmospheric pollen transport extends exposure beyond the local plant presence, so evolutionary shifts in flowering schedules and biomass are expected to reshape not only population spread but also the seasonal “shape” of the aeroallergen load experienced by downwind communities. Together, fecundity, seed-bank inertia, and long-distance dispersal can decouple local plant control from downwind exposure. This provides a mechanism by which pollen-season metrics can lag local suppression.

Long-term burial experiments further show that seed longevity depends heavily on source context, with seed lots from long-invaded, intensively managed arable systems maintaining very high viability after a decade of burial. In contrast, ruderal sources decline more sharply [32,46]. Alongside innate dormancy, this establishes a persistent temporal tail of recruitment that can support re-establishment after disturbance or incomplete control [5,31]. This seed-bank inertia explains why management aimed at suppressing adult biomass but not seed set and seed movement often results in only temporary gains. This raises the next mechanistic question: once introduced populations are established and genetically mixed, how quickly do they begin to track local climatic gradients through adaptive shifts in phenology and size?

### 4.2. Rapid Evolution of Flowering Phenology Shifts Pollen-Source Timing

A defining feature of *A. artemisiifolia* invasion is the repeated re-formation of latitudinal clines in flowering time and plant size after introduction. Clines observed in the native range have re-emerged in Europe and Australia within decades, consistent with strong climatic selection operating on abundant standing variation and admixture-generated recombination rather than waiting for de novo mutations [19,47]. Despite demographic bottlenecks during some introductions (e.g., in Australia), rapid adaptation has occurred through the reuse of pre-existing variants rather than de novo mutation [20,48]. Other latitudinal clines in flowering time/size have re-evolved independently in Europe/Australia within decades [20,49], while copy-number variation also contributes to adaptive divergence between historic/modern populations [50]. Genomic regions under selection are enriched for genes that regulate growth, defence and stress responses, phenology, and environmental tolerance [20,49]. Historic material adds temporal resolution by suggesting a two-stage trajectory: early spread can be supported by plasticity and broad tolerance, followed by rapid genotype–environment matching as locally advantageous variants increase in frequency [5,48,51,52,53,54]. Functionally, the selected genomic landscape maps to trait classes expected to co-determine establishment across heterogeneous habitats, as well as the timing, duration, and magnitude of pollen production. The remaining question is why these adaptive responses recur so consistently across invaded ranges, despite ongoing gene flow, an issue that is clarified by the structural genomic architecture now resolved at the chromosome scale.

For the present review, however, the important consequence is phenotypic rather than genomic: invaded populations can adjust reproductive timing to local climatic conditions and thereby shift when they become active pollen sources. Aerobiological monitoring and modelling indicate that climate change can lengthen *A. artemisiifolia* pollen seasons, particularly at higher latitudes, while scenario-based projections anticipate further increases in airborne pollen and sensitised populations across Europe [5,55]. Genetic mapping strengthens this interpretation by identifying an oligogenic architecture for phenological adaptation under high gene flow [20,56,57]. Genetic mapping and transcriptomic studies independently support the evolvability of flowering phenology [35,56]. Controlled-environment evidence showed that an invasive population flowered approximately 30 days earlier than the native-range comparator, while the subsequent male-maturation phase remained broadly unchanged [58]. Thus, adaptation can substantially advance reproductive onset without implying an equivalent extension of the atmospheric pollen season. This distinction is central to the plant–disease pathway because evolution can alter when pollen production begins, whereas the realized airborne exposure additionally depends on source abundance, pollen release, meteorology and atmospheric transport.

### 4.3. Quantitative Translation of Adaptive Traits into Aeroallergen Exposure

The genomic and phenological evidence discussed above establishes that adaptive divergence in *A. artemisiifolia* can be substantial, but plant-level trait differences should not be equated directly with human aeroallergen exposure. Translation across these scales involves at least three distinct quantities: the timing and magnitude of pollen production at the source, the fraction of that production reaching the atmosphere, and the biologically effective dose encountered by susceptible individuals. Available studies quantify individual links within this sequence, although they do not yet constitute a continuous genotype-to-clinical-outcome model.

At the source level, plant size provides one of the clearest quantitative links with pollen production [59]. Across natural French populations, seasonal pollen production ranged from approximately 0.1 to 3 billion grains per plant, depending on plant size and habitat [60]. Pollen output was strongly associated with dry biomass (r^2^ = 0.84) and plant volume (r^2^ = 0.73), with the biomass relationship described by log_10_ (pollen grains) = 7.22 + 1.12 log_10_ (plant dry biomass) [59]. As an illustrative calculation within the range represented by that dataset, a twofold difference in plant dry biomass corresponds to an approximately 2.17-fold difference in predicted pollen production [60]. This value should not be interpreted as a universal biomass-to-exposure conversion: stand density, resource availability, reproductive allocation, release efficiency, meteorology and atmospheric removal intervene between pollen produced by an individual plant and pollen measured in ambient air. Controlled temperature experiments similarly show that thermal conditions can substantially alter plant size and shift male flowering by ≈4–5 weeks, while final biomass and male raceme production do not necessarily change proportionally [33].

Phenological change presents a related but distinct problem. The approximately one-month difference in reproductive timing described in Section 4.2 demonstrates that adaptive divergence can markedly alter the timing of reproductive development; yet, the same experiment found no significant difference in the duration of the subsequent male-maturation phase [58]. An earlier reproductive transition therefore does not imply an equivalent extension of the atmospheric pollen season. At the regional scale, a 1995–2022 aerobiological series from the Milan area projected an *Ambrosia* pollen-season extension of approximately 6.5–7.8 days under temperature-driven scenarios [61]. Plant phenology and atmospheric pollen-season metrics therefore operate at different scales: the latter integrate many source populations, along with pollen release, transport, deposition, and meteorological removal.

Environmental forcing can additionally modify both the magnitude and biological composition of the pollen source. In controlled greenhouse experiments, doubling atmospheric CO_2_ from 350 to 700 μL L^−1^ increased stand-level ragweed pollen production by 61% [62]. In a separate experiment, pollen produced at 600 μmol mol^−1^ CO_2_ contained approximately 1.6-fold more Amb a 1 than pollen produced at 370 μmol mol^−1^, despite no corresponding increase in total pollen protein [63]. These estimates describe different components of exposure—pollen quantity and allergen content—and should not be multiplied to infer a combined increase in ambient Amb a 1. Their value lies instead in demonstrating that pollen-source magnitude and allergenic composition can vary independently.

At the continental scale, invasion and atmospheric processes can substantially amplify these local source changes. Hamaoui-Laguel et al. projected mean European airborne ragweed pollen concentrations to reach approximately four times contemporary levels by 2050, with an uncertainty range of approximately two- to twelvefold [64]. About one-third of the projected increase was attributed to continuing seed dispersal, whereas approximately two-thirds reflected climate and land-use effects, including increased pollen production in established populations under rising CO_2_ [64].

The final link to disease is supported by observational exposure–response data, although it remains population specific. In a Milan cohort of ragweed-allergic patients, airborne pollen concentration predicted conjunctival, nasal, and bronchial symptom severity. Study-specific median concentrations associated with progressively higher symptom classes were 1.33, 3.33, 14.5 and 50.67 pollen grains m^−3^ [65]. Taken together, these data support a staged rather than a direct relationship between adaptive evolution and allergic disease. Genomic variation can alter reproductive phenology and plant size; these traits influence the timing and potential magnitude of pollen production; atmospheric processes determine the realised airborne exposure; and host sensitisation and airway susceptibility determine its clinical expression. No study has yet linked adaptive genotype, reproductive phenotype, airborne pollen and Amb a 1 exposure, and longitudinal respiratory outcomes within the same source–population system. This missing cross-scale validation is a key limitation of the current evidence and an important priority for future research.

## 5. Climate-Modified Flowering, Airborne Pollen Exposure, and Allergen Delivery

**Figure 1:**
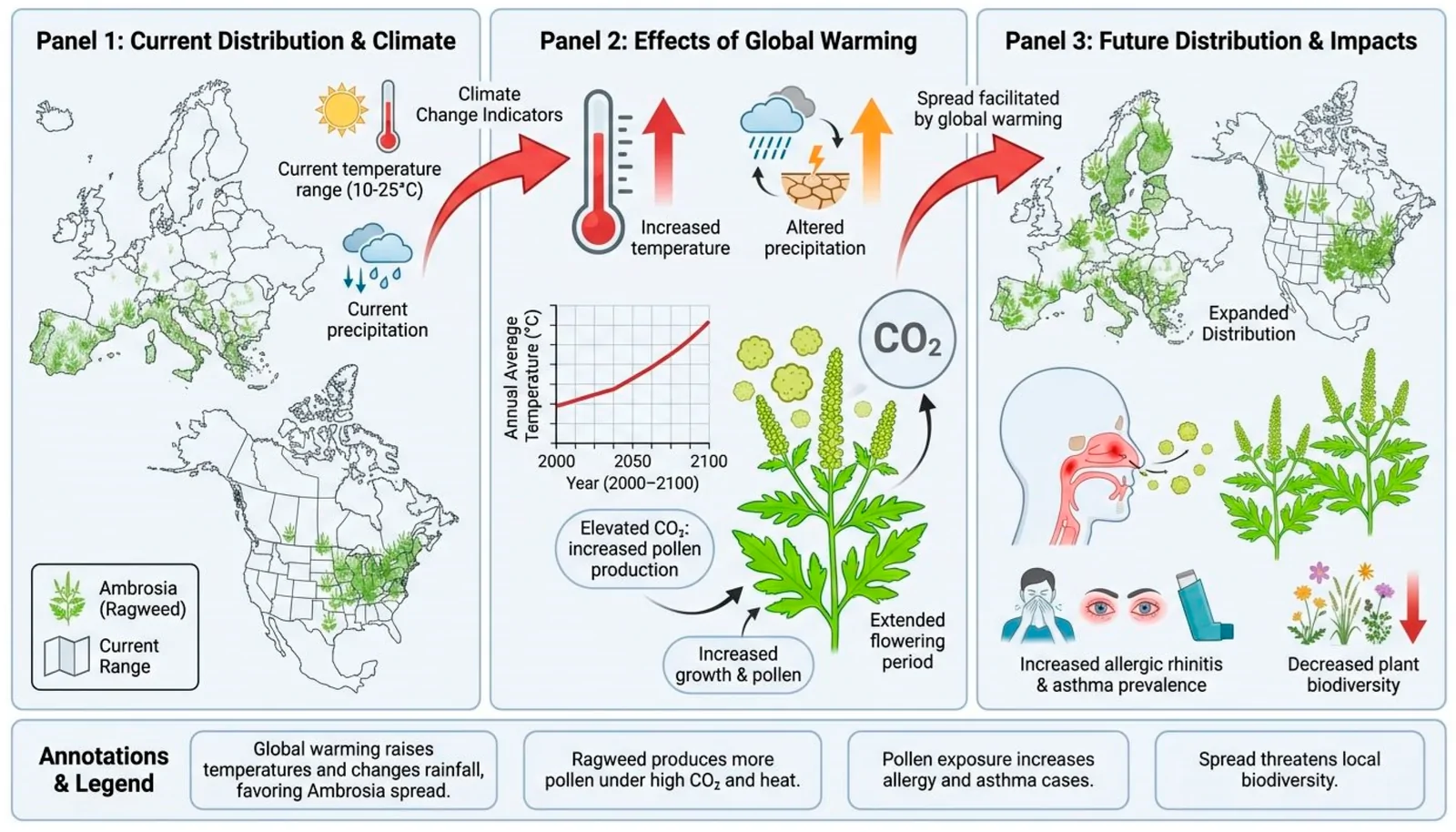
Climate-modified ragweed flowering, pollen production, and allergen exposure pathways. (**Panel 1**) summarises the present-day distribution and climatic envelope of *A. artemisiifolia*, emphasising that current occurrence reflects the combined effects of climatic permissiveness, anthropogenic habitat creation, repeated introductions, and atmospheric connectivity rather than a simple equilibrium niche. (**Panel 2**) illustrates the principal climate and atmospheric drivers expected to modify ragweed performance and exposure to global change, including warming, altered precipitation regimes, and elevated carbon dioxide (CO_2_), and their effects on growth, flowering phenology, pollen production, and allergenic potential. (**Panel 3**) depicts potential downstream consequences, including changes in geographic range, seasonal aeroallergen exposure, allergic respiratory burden and ecological pressure in invaded habitats. Arrows and spatial patterns represent the conceptual relationships synthesised in this review and should not be interpreted as quantitative effect sizes or as outputs from a single projection model.

The quantitative relationships described above define pollen-source potential rather than the inhaled aeroallergen dose itself. Between pollen production and human exposure lie several atmospheric filters, including source density, release efficiency, meteorological transport, deposition and washout, pollen rupture, allergen content per grain, and the aerodynamic size of allergen-bearing material [33,63,66]. This distinction is particularly important for *A. artemisiifolia*, because atmospheric Amb a 1 concentrations and pollen allergen potency can diverge from intact pollen counts. The following section therefore examines how changes at the plant source are transformed into temporally and spatially heterogeneous airborne exposure. As summarised in Figure 1, future ragweed risk should be interpreted as the outcome of interacting climatic, atmospheric, ecological, and clinical processes that connect present distribution with altered pollen production, allergen delivery, and downstream health burden.

### 5.1. Aerobiological Monitoring and Exposure Metrics

Standard aerobiological monitoring remains the cornerstone of ragweed exposure assessment because it provides harmonised, long-term, taxonomically resolved data on airborne pollen, including identifiable pollen grains and identifiable pollen fragments. In Europe and other allergy-monitoring networks, the volumetric Hirst-type method, standardised in European Standard (EN) 16868:2019 [67], issued by the European Committee for Standardisation (CEN), is widely used for continuous sampling and microscopic analysis of airborne pollen and fungal spores [68]. These data underpin pollen calendars, exposure mapping, forecasting, epidemiological studies, and public-health warning systems. Therefore, pollen concentrations should not be regarded as an inadequate measure, but as the essential reference layer on which more detailed exposure metrics can be built. At the same time, clinically relevant ragweed exposure may not always be fully captured by the measured pollen concentration signal alone, because atmospheric processing can alter allergen release, particle-size distribution, and the relationship between microscopy-based pollen concentration estimates and inhaled allergen dose. During atmospheric transport, pollen grains and fragments encounter fluctuating circulation patterns, humidity, rainfall, and chemical stressors; under such conditions, rupture or cytoplasmic extrusion through apertures can release allergen-containing material into the air [10,69,70]. Experimental evidence further indicates that oxidative and nitrative chemistry can modify ragweed antigenicity, including changes in Amb a 1 antigenicity and oxidative modifications such as nitration, nitrosylation, and carbonylation [60]. Once released, allergenic components associated with smaller particles may have lower sedimentation velocities than larger pollen particles, remain airborne longer, disperse farther, and penetrate more deeply into the respiratory tract. Accordingly, in this review, *A. artemisiifolia* aeroallergen exposure is treated as a layered exposure system comprising: (i) standard aerobiological pollen concentrations, derived from identifiable pollen grains and identifiable fragments; (ii) respirable allergen-bearing fractions, including subpollen particles (SPPs) and allergen associated with fine aerosol particles; and (iii) molecularly measured airborne allergen load, such as Amb a 1 concentration and pollen allergen potency. This integrated framing preserves the central value of pollen monitoring while explaining why complementary allergen- and particle-resolved measurements can improve interpretation of biologically effective dose and lower-airway risk under specific meteorological and pollution conditions.

This distinction becomes especially informative when concentrations of pollen particles and airborne allergens are measured simultaneously. A recent atmospheric monitoring study in Bratislava operationalised co-measurement of airborne ragweed pollen and Amb a 1 allergen by pairing a Hirst-type pollen trap (microscopy) with cyclone sampling and enzyme-linked immunosorbent assay (ELISA) quantification of Amb a 1 (pg/m^3^). This allowed calculation of pollen allergen potency (PAP; pg Amb a 1 per pollen) and identification of meteorological and pollutant correlates, including relative humidity as a negative correlate of allergen levels and carbon monoxide (CO) and sulfur dioxide (SO_2_) as relevant pollutant signals in regression models [10]. Mechanistically, this matters because it shows that pollen concentration and airborne allergen load can decouple, particularly when long-distance transport, humidity-linked release processes, and pollutant chemistry modify allergen availability [10]. Complementary synthesis on nitrogen pollution and pollen allergy likewise emphasises that atmospheric pollutants often co-vary, complicating causal attribution and supporting multi-pollutant designs and integrated exposure metrics when assessing ragweed allergenicity under field conditions [71].

High ragweed pollen episodes should not be interpreted only as exceedances of a fixed symptom-onset threshold. In Bratislava, daily airborne *A. artemisiifolia* pollen concentrations reached approximately 500 pollen/m^3^, illustrating that invaded urban and peri-urban landscapes can generate intense exposure episodes [10]. However, recent evidence from the EPOCHAL (Effect of Pollen on Cardiorespiratory Health and Allergies) study indicates a nonlinear pollen exposure–symptom relationship, with no clear concentration below which symptoms are absent and with attenuation of symptom increase beyond approximately 40–80 pollen/m^3^ [72]. The same study showed that recent exposure, particularly during the preceding few hours, contributed strongly to symptom severity, while effects could persist across several days. Ragweed exposure should therefore be interpreted through a dynamic exposure–response model shaped by pollen concentration, recent and cumulative exposure, individual sensitisation, meteorology, air pollution, and allergen bioavailability. Within Europe, recurrent regions such as the Great Pannonian Plain, the Po Plain, and the Rhône Valley are consistently associated with high pollen levels, reflecting invasion history alongside disturbance- and agriculture-related landscapes that support establishment, persistence, and spread [5].

Pan-European pollen monitoring syntheses indicate that some of the highest airborne *Ambrosia* pollen levels occur in regions such as France, northern Italy, the Pannonian Plain, and Ukraine, with spatial gradients away from these centres and locally variable trends reflecting changing source strength, distance, and management or biocontrol effects [73]. From a health perspective, “distribution” is partly an atmospheric construct: long-distance transport creates exposure far from source stands, so the climatic envelope of plant establishment differs from that of population exposure [28]. Transport-modelling studies, trajectory and dispersion algorithms already demonstrate clear source–receptor connectivity across Europe [28]. A major open question is how climate-driven changes in circulation will alter the frequency, intensity, and timing of such transport episodes and how these changes should be incorporated into burden projections and warning thresholds [28].

Pre-season southerly airflow transported pollen and allergenic proteins from Hungary, where the season begins earlier, and specific weather regimes were associated with relatively higher levels of non-pollen-bound allergens compared with pollen grains [5,10]. Post-season conditions, often wetter and windier, were associated with elevated levels of pollen-associated allergen particles, consistent with multiple non-exclusive mechanisms, including delayed pollen release, osmotic rupture during rain events, and mechanical rupture under stronger winds [10]. A controlled-environment study provides plausible mechanistic intermediates for potency shifts, reporting that elevated temperature and nitrogen dioxide (NO_2_), particularly in combination, were associated with increased oxidative stress indicators and higher allergenicity, while NO_2_ also altered pollen flavonoid composition, which was dominated by quercetin-class compounds [74]. Direct aerobiological evidence of microscopic fungi and other contaminants on airborne ragweed pollen further supports the view that the inhaled exposure mixture may extend beyond pollen alone and provides a rationale for incorporating microbial or epiphytic profiling into allergen surveillance [17].

Clinically relevant exposure is anchored in standard airborne pollen monitoring, but symptom burden may depend additionally on allergen dose, allergen bioavailability, particle size, and atmospheric processing. Across the literature, pollen potency varies across space and time, and its drivers remain incompletely resolved; however, ragweed-specific time-series show that seasonal pollen totals may remain relatively stable while Amb a 1 season integrals and potency vary substantially, implicating drought, sunshine, humidity, and pollutant lags [10,75,76]. Moreover, allergen-bearing particles occur in respirable fractions, including particulate matter with aerodynamic diameters ≤10 μm (PM_10_) and ≤2.5 μm (PM_2.5_) and post-peak persistence, providing a mechanistic basis for symptom burden and lower-airway risk that may diverge from airborne pollen concentrations alone [77]. Long-distance transport further extends this exposure system by generating clinically relevant episodes in regions without established ragweed populations, thereby adding episodic atmospheric risk to local seasons [6,28,78]. Future health burden should therefore be understood as the product of climate- and land-use-driven changes in pollen load, spread dynamics, exposure pathways, and sensitisation patterns, with projected burden remaining highly sensitive to invasion control as an adaptation strategy [64,70].

Meteorology, therefore, acts jointly on emission, transport, and transformation. Warm, sunny weather promotes pollen maturation and anther opening, thereby supporting a positive association between temperature and airborne pollen concentrations. Pollen allergen potency (PAP) has also been described as temperature-sensitive, consistent with evidence that temperature and pollutant context can modify pollen traits and allergenic potential [10,74,79]. By contrast, precipitation and high humidity can suppress airborne pollen through washout and inhibit anther opening, whereas wind functions as a multi-purpose amplifier by drying or stressing anthers, lifting and dispersing pollen, resuspending deposited material, and enabling long-range transport under favourable synoptic patterns [10,79]. Particle size adds a final layer of complexity, because smaller allergen-bearing fractions can persist longer and penetrate deeper into the respiratory tract, meaning that exposure severity may increase even when intact pollen concentrations do not rise proportionally.

### 5.2. Climate and Atmospheric Drivers of Growth, Phenology, Pollen Production, and Allergenic Potential

Controlled-environment studies indicate that ragweed exhibits broad temperature tolerance together with temperature-responsive changes in growth, flowering, and pollen allergenicity. Importantly, allergenicity may increase with temperature through changes in allergen synthesis and Amb a 1 IgE-binding [33]. This implies that warming can influence not only biomass and phenological success, but also the per-grain biological activity of exposure. CO_2_ enrichment provides an even stronger mechanistic link between atmospheric change and clinical risk. Elevated CO_2_ increases reproductive output and pollen production and, under some exposure regimes, increases the content of major clinical allergens without changing total pollen protein [62,80,81]. More importantly, pollen produced under elevated CO_2_ has been shown to induce stronger allergic lung inflammation than pollen from control plants, demonstrating that plant growth conditions can alter clinically relevant inflammatory potential rather than merely increasing pollen quantity [81]. Collectively, these findings support a mechanistic interpretation in which CO_2_ can scale both the quantity and the quality of ragweed exposure [63].

By contrast, evidence for other pollutants remains more mixed and should be interpreted more cautiously. NO_2_ enhances allergenicity through protein nitrosylation, supporting a direct molecular pathway from traffic-related pollution to altered pollen bioactivity [82]. Ozone, in contrast, alters the transcriptome but does not consistently increase total Amb a 1 protein [83]. The current evidence, therefore, supports pollutant-specific rather than uniform effects of pollution. An important research priority is to quantify how allergen dose and potency respond under realistic multi-factor conditions that combine CO_2_, heat waves, drought, and pollutant mixtures, using linked endpoints such as pollen production, Amb a 1 content, post-translational modification, allergen localisation, and immunoreactivity.

Rainfall responses further reinforce that risk cannot be inferred from warming alone. Rainfall-manipulation experiments show that fitness responses to reduced rainfall are spatially structured, with population-by-environment interactions indicating that historical climate and geography shape responses to future drying and extreme precipitation regimes [66]. Aerobiological allergen measurements complement these plant-level responses: in a multi-year time series, drought and sunshine were associated with higher seasonal pollen integrals, while August drought was implicated in higher Amb a 1 season integrals and higher pollen allergen potency, suggesting that water stress can alter allergen allocation in clinically relevant ways even when seasonal pollen totals are similar [10,76]. The field now has demonstrated methods for measuring and so the translational challenge is therefore not simply to forecast pollen but to build exposure metrics that better predict symptoms and exacerbations than standard pollen concentration data alone.

### 5.3. Future Airborne Exposure Scenarios and Remaining Modelling Uncertainties

Modern projections indicate that future airborne ragweed pollen concentrations in Europe may increase substantially by mid-century, with estimates of around a fourfold increase by 2050 under some scenarios, although uncertainty remains large and is strongly driven by assumptions about seed dispersal and spread [64]. Species-range projections similarly suggest northward and eastward expansion of suitable conditions in Europe and expansion of high-allergy-risk areas under climate scenarios. In contrast, eastern North America may experience northern expansion but contraction in some southern regions, underscoring that future risk is spatially heterogeneous rather than uniformly increasing everywhere [84,85]. A further key result is that sensitisation may more than double in Europe under mid-century scenarios, again with strong dependence on invasion and spread assumptions, implying that management can materially alter the public-health trajectory [62].

The most defensible pathway from future pollen to future disease is through exposure metrics that capture allergens, symptom thresholds, and transport-aware episodes rather than relying on pollen concentration alone. High-resolution mapping already indicates a large current burden concentrated in known pollen hotspots [70]. Cohort evidence from the Milan region supports that ragweed pollen load predicts ocular, nasal, and bronchial symptom intensity, and shows that correlations and thresholds vary and may be modified by immunotherapy [65]. Long-distance transport can also be clinically relevant in ostensibly low-risk settings: high-altitude sites may experience extreme short-term pollen loads from remote sources, together with high symptom reporting among sensitised individuals, strengthening the rationale for cross-border monitoring and transport-aware forecasting [77]. Ragweed pollen is known to travel over very long distances, including episodes of >1000 km, reinforcing the need for future burden models to resolve transport as well as local establishment [28].

Future ragweed threat is best treated as an emergent systems problem in which clinically relevant exposure is an atmospheric mixture of intact pollen, subpollen particles, pollutants, and allergen-bearing fine aerosols whose biological activity varies with CO_2_, temperature, drought, and pollution. This substructure explains why pollen concentration alone can misestimate risk, why long-distance transport can generate remote exposure episodes, and why future burden cannot be inferred from climate suitability alone. The most urgent next step is to link invasion dynamics, airborne allergen measurements, symptom-resolved surveillance, and patient-level outcomes within a shared predictive structure that can improve burden projections and guide spread control, land-use management, and climate-health adaptation.

## 6. Ragweed Sensitisation, Molecular Allergens, Cross-Reactivity, and Clinical Relevance

Ragweed sensitisation should be interpreted as a clinical and epidemiological endpoint, not simply as a mirror of plant distribution. The presence of *A. artemisiifolia* in a region creates the ecological basis for exposure. Still, measured sensitisation depends on several additional layers: pollen load, season length, local abundance of source populations, atmospheric transport, co-exposure to other weed pollens, air pollution, urban land use, diagnostic access, and the type of allergological testing used [10,24,80,86,87,88,89]. This distinction is essential because sensitisation, clinically relevant allergy, and respiratory disease burden are not equivalent outcomes. Extract-based IgE positivity or skin-prick-test reactivity indicates immunological recognition. In contrast, clinically relevant ragweed allergy requires concordance between exposure, sensitisation profile, symptoms, medication use, and, where applicable, lower-airway involvement.

### 6.1. Regional Sensitisation Patterns and Clinical Burden

Europe provides the clearest example of how long-established invasion can generate a marked hotspot–gradient structure of sensitisation burden. Approximately 16 million Europeans are estimated to be sensitised to *Ambrosia*. Still, this burden is highly uneven, remaining below 5% in some low-exposure areas while exceeding 20% in major hotspots such as Hungary [21]. This pattern reflects sustained exposure in long-established invasion corridors. The highest burdens occur in long-established exposure corridors such as the Pannonian Plain, the Po Valley, the Rhône Valley, and parts of the Balkans, where dense invaded stands, recurrent late-summer pollen pressure, and repeated seasonal clinical exposure converge [5,6,21,22]. Hungary and neighbouring regions repeatedly emerge as the European epicentre, while Szeged is frequently used as a reference site in aerobiological analyses under high-exposure conditions [21,90]. In contrast, Atlantic and Nordic Europe generally remain lower-prevalence settings, often below 5–8%, consistent with weaker establishment histories and less sustained exposure. However, continued spread and climate change may modify this pattern [6,21,23].

The European case also shows why crude regional averages can be misleading. An often-cited “approximately 10%” average hides a steep gradient from Atlantic and Nordic low-exposure regions to Central and Eastern European and river-valley corridors [6,10,21,23,24]. A broad intermediate zone extends across parts of Central and Southern Europe, where allergic-cohort estimates generally fall between the major hotspot corridors and the low-exposure Atlantic/Nordic fringe. However, direct comparison among regions remains difficult because studies differ in cohort composition, recruitment setting, diagnostic method, allergen extract, component availability, and whether sensitisation is assessed in the general population or among symptomatic allergic patients [21,22,23,86]. Therefore, the European sensitisation map should be read as the product of both biological exposure and diagnostic architecture.

The clinical importance of this gradient is considerable. Europe-wide estimates suggest that approximately 13.5 million individuals have clinically relevant *Ambrosia*-related allergies, with an annual economic cost of approximately €7.4 billion [21,70]. These figures demonstrate that ragweed sensitisation is not only an indicator of invasion success, but also a measurable public-health burden. Nevertheless, the difference between sensitisation and symptomatic disease must remain explicit. A region may show IgE positivity without a proportional level of clinically relevant ragweed allergy if exposure is intermittent, if cross-reactivity inflates test positivity, or if the tested population is not representative of the general population. Conversely, high-exposure regions may produce a stronger link between sensitisation and symptoms because exposure is repeated, seasonal, and intense.

Future European projections strengthen this concern. Current quantitative estimates focus mainly on Europe and suggest that the number of sensitised individuals may increase from around 33 million to roughly 77 million by 2041–2060 [55]. These projections should not be explained as a simple consequence of climate warming alone and depend on assumptions about future spread, pollen production, exposure intensity, land-use disturbance, population distribution, and the effectiveness of control strategies. The absence of comparable continent-wide estimates for Africa in the same modelling structure also highlights an important imbalance in global evidence [55]. Thus, Europe is currently the best quantified system, but not necessarily the only region where the burden may increase.

Regional case studies help clarify why sensitisation estimates require careful interpretation. In Slovakia, comparisons between urban and rural areas showed similar *A. artemisiifolia* pollen and allergen levels during the late-summer and autumn flowering period, suggesting that once regional exposure is widespread, simple urban–rural contrasts may not capture true exposure differences [5,86]. This is relevant clinically because patients may experience comparable seasonal exposure even when local microenvironments differ. However, the Slovakian example should not be overextended into a general rule; it mainly shows that local setting, regional source strength, and atmospheric mixing may interact in ways that complicate exposure attribution.

Switzerland provides a different, clinically important example. In a national adult cohort, 7.9% of participants had *A. artemisiifolia* species-specific IgE, but only 1.3% were monosensitized, while 82% of those sensitised to *A. artemisiifolia* were also sensitised to *Artemisia* pollen [91]. This finding shows that in lower-exposure or mixed weed-pollen settings, apparent ragweed sensitisation may not always represent primary ragweed allergy. Instead, it may reflect cross-reactivity, co-sensitisation, or diagnostic overlap between *Ambrosia* and *Artemisia*.

The Ankara example adds another layer. Aerobiological studies in central Anatolia indicate that transported pollen and Amb a 1 can affect local exposure, with inferred source regions including continental Ukraine, Crimea, and Russia [78]. Clinically, this means that the burden of ragweed sensitisation may not always be explained by local plant populations alone. Long-distance transport can create exposure episodes in regions where local establishment is limited or where external source areas influence the pollen season. For clinical interpretation, this reinforces the need to link patient data with exposure history and regional aerobiological information rather than assuming that sensitisation reflects only local distribution.

North America remains the benchmark for populations exposed to ragweed over long periods and at high intensity. In the United States, general population sensitisation has been estimated at 26.2% in the Third National Health and Nutrition Examination Survey (NHANES III), providing a reference point for comparison with invaded regions where exposure is more recent, spatially heterogeneous, or diagnostically less standardised [21,33]. In Canada, a multi-site study reported general population sensitisation of approximately 15.3%, placing Canada below the United States but above many European countries and most Asian contexts in the cited comparison [21]. These North American values are useful because they represent regions where ragweed exposure is historically established and where sensitisation has developed under sustained ecological and atmospheric pressure.

Compared with North America and Europe, the Australian clinical and aerobiological burden remains less comprehensively characterised. Nevertheless, evidence from the Northern Rivers region of New South Wales documented a ragweed pollen season from early March to early May, a peak daily concentration of 483 pollen grains/m^3^, and 34% skin-prick-test positivity to ragweed among tested volunteers [92]. This finding is important because it demonstrates that clinically relevant regional exposure can occur in Australia. However, it should not be generalised into a continent-wide burden. The Australian case is best interpreted as a geographically restricted but clinically meaningful example, where regional pollen exposure and sensitisation have been documented, while broader national patterns remain insufficiently characterised.

Across Asia, ragweed is not consistently the dominant pollen allergen, and reported sensitisation is generally lower than in Europe and North America. However, regional heterogeneity is substantial. Korea has reported an 8.7% ragweed sensitisation rate in a national survey. In contrast, Japanese pediatric allergy data indicate that sensitisation rates in children can exceed 30%, showing how age structure, sampling strategy, and diagnostic framing can influence reported prevalence [21,93]. These examples indicate that Asian ragweed allergy cannot be reduced to a single continental pattern. Instead, sensitisation depends on regional invasion history, co-dominant weed pollens, patient selection, and diagnostic approach.

China is especially important because it illustrates the difficulty of separating true ragweed allergy from mixed weed-pollen sensitisation. Since its first detection in northeastern China in 1935, *A. artemisiifolia* has been reported in more than 20 provinces, and its clinical relevance is increasing among patients with allergic rhinitis and asthma [24]. Sensitisation to *A. artemisiifolia* among Chinese allergic populations ranges from approximately 3.7% to 14.8%, predominantly in northern cities, with examples including Beijing at 14.68%, Taiyuan at 19% among allergic rhinitis patients, Tangshan at 3.7%, and Dongying at 14.78% [21,94]. However, these values require cautious interpretation because many Chinese patients show co-sensitisation with Artemisia species, and cross-reactive carbohydrate determinants (CCDs) can further complicate extract-based IgE results [95,96,97]. Consequently, part of what appears to be *A. artemisiifolia* positivity, especially in southern and western China, may reflect cross-reactivity rather than true primary ragweed allergy [95,96,97].

The African evidence base is much thinner. Available data are concentrated mainly in South Africa, where a national aeroallergen monitoring and molecular allergy study reported 8.2% sensitisation to *Ambrosia* among tested allergic patients in Cape Town using an Allergy Explorer (ALEX) array [88]. A second study in the industrial Vaal Triangle found 10.3% sensitisation to *A. artemisiifolia* by skin-prick testing among atopic adults [89]. Together, these findings suggest that ragweed may be a previously under-recognised aeroallergen in parts of sub-Saharan Africa. However, the evidence remains almost entirely dependent on recent South African studies, with little quantified prevalence information from other African countries. Africa should therefore be described as a region of measurable but still poorly mapped burden, not as a continent with an already defined ragweed-sensitisation pattern.

Across regions, several interpretive principles emerge. First, ragweed sensitisation is most consistently elevated where long-established invasion corridors generate sustained seasonal exposure. Second, measured prevalence is strongly affected by study design, tested population, diagnostic platform, and access to specialist allergy services. Third, co-dominant weeds, especially Artemisia, can inflate apparent ragweed sensitisation through cross-reactivity or co-sensitisation [87,91,98]. Fourth, air pollution, urban disturbance, climate-related changes in pollen production, and environmental stressors may modify allergenicity or symptom expression, meaning that pollen abundance alone is an incomplete predictor of clinical burden [10,24,60,77,80,81,82,86,99].

The clinical implication is that ragweed sensitisation should be interpreted as a layered principle. At the first layer, invasion and pollen production determine the opportunity for exposure. At the second layer, atmospheric transport, season length, pollen load, pollutants, and environmental stressors modify the dose and biological quality of exposure. At the third layer, the host response depends on molecular sensitisation profile, cross-reactivity, co-sensitisation, and individual susceptibility. Only at the final layer does sensitisation translate into clinically relevant rhinitis, rhinoconjunctivitis, asthma symptoms, medication use, or exacerbations. This layered interpretation prevents overstatement of prevalence data and sets up the following subsection, where Amb a 1, minor allergens, cross-reactive molecules, and component-resolved diagnostics are considered tools for distinguishing genuine ragweed allergy from broader weed-pollen sensitisation.

Taken together, the available evidence supports a regional but not uniform global pattern. Europe represents the best-defined hotspot–gradient system; North America remains the benchmark for sustained high-intensity exposure; Asia is heterogeneous and frequently complicated by overlap with *Artemisia*; Australia provides a geographically restricted but clinically relevant example; and Africa remains measurable but poorly mapped [21,30,88,89,91,92,93,94,98]. The key unresolved issue is not whether ragweed can induce sensitisation, but how accurately current surveillance systems distinguish true *A. artemisiifolia* allergy from extract positivity, cross-reactivity, and mixed weed-pollen exposure.

### 6.2. Molecular Allergens, Cross-Reactivity, and Clinical Attribution

The clinical interpretation of ragweed sensitisation depends on identifying which allergen molecules drive the IgE response. Component-resolved allergology can distinguish genuine *A. artemisiifolia* sensitisation from cross-reactivity with other weeds and broader panallergen-driven IgE binding. Amb a 1 remains the dominant source-associated marker, with IgE reactivity reported in more than 90% of ragweed-allergic patients in several cohorts, although estimates vary with assay and population [100,101]. The repertoire is broader, however, and includes clinically relevant components such as Amb a 6, Amb a 8, Amb a 9, and Amb a 10, whose contribution is more context-dependent [21,100,101]. Amb a 11 adds a mechanistically distinct major allergen because of its cysteine-protease activity [102,103], whereas Amb a 4 and Amb a 12 are particularly relevant to cross-reactive or syndrome-specific interpretation [87,104].

Recombinant Amb a 1.01, with allergenic properties close to the natural molecule, also provides a better-defined reagent for molecular assays and standardisation [105]. Molecular attribution should be separated from exposure intensity. Extract-based IgE can overstate source-specific sensitisation when profilins, polcalcins, cross-reactive carbohydrate determinants (CCDs), or overlapping *Artemisia* components dominate the response [106,107]. Conversely, a source-specific molecular profile does not by itself define the inhaled dose: airborne Amb a 1 and pollen allergen potency can diverge from intact pollen counts because allergen content, atmospheric processing, and respirable allergen-bearing fractions vary with environmental conditions [10,74,76,108]. Component-resolved diagnostics and atmospheric allergen measurements therefore answer complementary questions: who is genuinely sensitised, and what biologically relevant material is being inhaled? They are most informative when interpreted together rather than treated as substitutes for conventional aerobiological monitoring.

For clinical attribution, the ragweed repertoire can be organised into five functional classes. Class I comprises source-associated major allergens, principally Amb a 1 and Amb a 11. Class II includes context-dependent protein allergens that refine endotypes or cross-reactivity, including Amb a 4, Amb a 6, Amb a 8, Amb a 9, Amb a 10, and lower-frequency components such as Amb a 3, Amb a 5, and Amb a 7; Amb a 12 is best regarded as a cohort- and assay-dependent panallergen candidate [21,106,109,110]. Class III captures glycan-related IgE binding, which can generate positive extract-based tests with uncertain symptom relevance [107]. Class IV represents environmentally modulated pollen potency, i.e., variation in allergen content and biochemical context per grain [74,106], with ragweed-relevant experimental evidence linking elevated temperature and NO2 exposure to increased allergenic potential and altered pollen biochemistry. Class V represents the physical form of exposure, including subpollen particles (SPPs) and other respirable allergen-bearing fractions that can alter lower-airway delivery independently of intact pollen concentration [108,111].

The diagnostic value of these classes is not equivalent. Classes I-III primarily address source attribution, whether the IgE signal is genuinely ragweed-specific or confounded by cross-reactive proteins or glycans. In contrast, Classes IV and V describe properties of the exposure itself. This distinction prevents a common interpretive error: a patient may have highly specific Amb a 1 or Amb a 11 sensitisation but could encounter a low atmospheric allergen dose. In contrast, another patient may experience intense airborne exposure yet show an IgE profile dominated by cross-reactive components. Molecular phenotype and exposure phenotype should therefore remain analytically separate until linked by clinical outcomes. Importantly, the proposed five-class structure has not yet been empirically validated in an independent cohort and should therefore not be interpreted as a diagnostic algorithm or a validated clinical prediction model.

Table 1 summarises recognised *Ambrosia* allergens by molecular family, reported IgE reactivity, and diagnostic relevance. Reported frequencies should be interpreted as assay- and cohort-dependent rather than as universal prevalence values [10,107,112,113].

Table 1 should therefore be interpreted primarily as a guide to molecular attribution rather than as a hierarchy of clinical severity. Amb a 1-dominant profiles support genuine *A. artemisiifolia* sensitisation, whereas profiles dominated by profilins, polcalcins, CCD-related IgE binding, or overlapping *Artemisia* components require greater caution because they may reflect cross-reactivity or mixed weed-pollen sensitisation [106,107]. Component-resolved profiling can accordingly support source attribution, cross-reactivity assessment, and interpretation of ragweed-directed immunotherapy, while clinical relevance remains anchored to exposure history and symptom concordance [104,119,120].

## 7. Mechanisms of Ragweed-Induced Airway Inflammation

*A. artemisiifolia* pollen allergy exemplifies how a seasonally recurring aeroallergen can cause significant inflammation across the respiratory mucosa, manifesting as seasonal allergic rhinitis/rhinoconjunctivitis and, in a clinically relevant subset of sensitized individuals, lower-airway symptoms consistent with allergic asthma or asthma exacerbations. In temperate regions, symptoms peak during the late-summer and early-autumn pollen season, with cough, wheeze, and dyspnoea concentrated in patients with asthma, bronchial hyperresponsiveness, or overlapping airway disease [115,121,122]. Although the downstream IgE-dependent type 2 response is broadly shared with other seasonal pollen allergies, ragweed provides a particularly well-characterized model in which allergen composition, respirable pollen-derived particles, protease activity, oxidative signaling and low-molecular-weight pollen cofactors modify the airway context in which that response develops.

**Figure 2:**
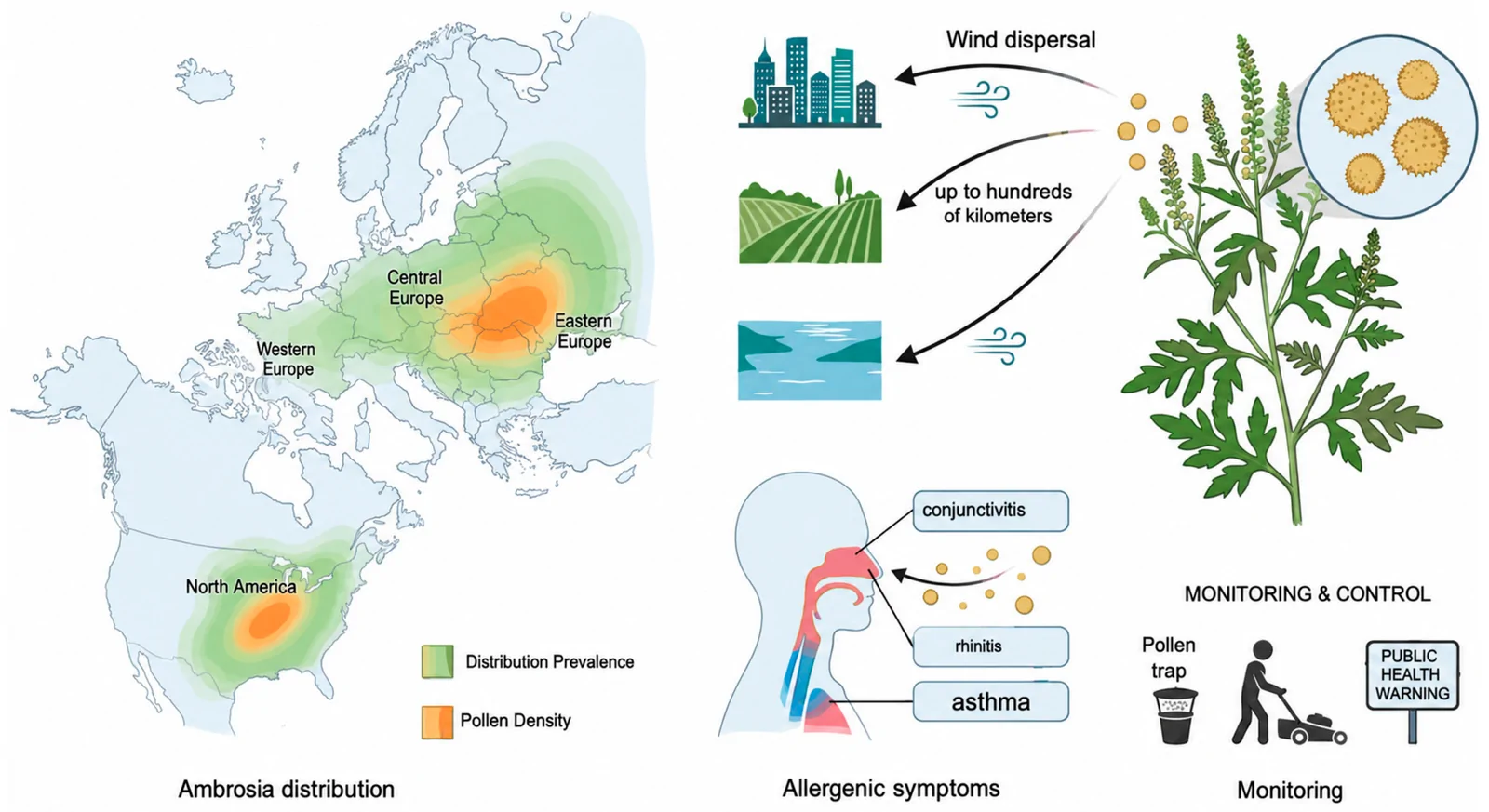
Conceptual pathway linking *A. artemisiifolia* distribution, pollen dispersal, allergic symptoms, and public-health monitoring. The ecological component illustrates established source regions and areas of comparatively high ragweed burden, with North America representing the native range and Central and Eastern Europe, including the Pannonian region, representing an important invaded high-exposure area [21]. Wind-mediated dispersal illustrates how airborne pollen can generate exposure beyond local source populations. The clinical component links inhaled exposure with upper- and lower-airway manifestations, including conjunctivitis, rhinitis and asthma, whereas the surveillance component represents pollen monitoring, public-health communication and source-control measures. The geographic shading and pollen-density patterns are schematic and are intended to illustrate relative source and exposure concepts; they do not constitute a quantitative prevalence map or measured pollen-concentration dataset.

Figure 2 provides a conceptual overview of ragweed as a coupled distribution–dispersal–exposure–health system, linking source regions and pollen transport with respiratory manifestations, surveillance, and control.

### 7.1. Shared Type 2 Effector Pathway: Direct Evidence from Human Ragweed Challenge

Human airway-challenge studies provide direct evidence that the canonical type 2 pathway is engaged during ragweed-induced lower-airway inflammation. Segmental ragweed challenge increased IL-13 transcripts and IL-13 secretion in bronchoalveolar lavage from atopic patients, while earlier challenge studies linked IL-5, but not RANTES, to eosinophil recruitment and degranulation [123,124].

In a separate study of six patients with mild asthma, ragweed challenge increased bronchoalveolar IL-5 to 984 ± 588 pg mL^−1^ compared with 2.8 ± 0.2 pg mL^−1^ at saline-challenged sites; IL-5 correlated strongly with eosinophil recruitment (r = 0.90) and eosinophil-derived neurotoxin release (r = 0.89), whereas RANTES did not correlate with either outcome [124]. Additional earlier evidence of local allergen-specific immunity comes from 19 ragweed-allergic asthmatic subjects studied before and after segmental lung challenge. Ragweed-specific IgE, IgA and IgG were detectable in bronchoalveolar lavage, and allergen-specific antibody responses were associated with eosinophil cationic protein, supporting local allergen-specific immune activity within the lower airway [125].

The best-established effector pathway is IgE-mediated type I hypersensitivity. Airway exposure promotes allergen uptake by dendritic cells, type 2 helper T-cell differentiation, B-cell class switching to allergen-specific IgE, and recruitment of eosinophilic inflammation [121,126,127,128]. Type 2 immunity remains central to allergic airway inflammation. In sensitised individuals, group 2 innate lymphoid cells (ILC2s) and type 2 helper T cells secrete interleukin (IL)-4, IL-5, and IL-13, thereby promoting eosinophilia, IgE class switching in B cells, mucus hypersecretion, and tissue remodelling. Upon allergen re-exposure, multivalent allergen molecules cross-link IgE-FcεRI complexes (pre-)occupying the surface of mast cells (or basophils), leading to activation and degranulation [129,130,131,132,133,134,135]. Epithelial alarmins, particularly IL-25, IL-33, and thymic stromal lymphopoietin (TSLP), sit upstream of this response by conditioning dendritic-cell function and activating type 2 helper T cells and ILC2s [132,134]. More generally, allergen-induced epithelial stress can regulate IL-33 release through mechanisms involving stress-granule assembly and gasdermin D fragmentation [136]; however, these pathways have not been shown to distinguish ragweed from other pollen allergies. Emerging evidence further implicates immune training, neuroimmune circuitry, and macrophage plasticity in disease expression, while environmental and nutritional exposures may exert particularly important effects during the perinatal and early-life periods [131,137,138,139,140].

Previous human challenge data support the clinical relevance of this axis. These findings place allergen-specific type 2 immunity at the centre of established human disease. Several feedback circuits can then modify its intensity: FcεRI-expressing nociceptors can further amplify allergic inflammation by releasing neuropeptides that promote the influx of type 2 helper T cells, creating a feedforward loop that may intensify disease severity [131]. Eosinophils reciprocally enhance ILC2 function, and alternatively activated (M2) macrophages may amplify ILC2-driven inflammation via extracellular vesicles [134,141,142]. Others have also suggested that dietary context, including high-fructose intake, can aggravate airway reactivity or modify pollen allergenicity in murine models [143]. These modifiers are biologically relevant but should not be assigned the same evidential weight as the IgE–FcεRI/type 2 axis, because much of their support is experimental or indirect. The clinical effectiveness of biologics directed against IgE, IL-5/IL-5R, IL-4Rα, and TSLP is consistent with the importance of the central type 2 pathway, while incomplete responses indicate that upstream exposure and tissue-level amplification remain clinically consequential [144,145,146].

### 7.2. Epithelial and Innate Amplification at the Exposure Interface

Unlike the shared downstream type 2 pathway described above, the following mechanisms concern properties experimentally demonstrated for the ragweed pollen exposure itself. Ragweed pollen is delivered to the airway not as allergen protein alone but as a complex biological matrix whose particle size, enzymatic activity, oxidative capacity and innate signaling potential can modify the local response. Hydrated pollen can release respirable subpollen particles (SPPs) carrying allergenic proteins and functional reduced nicotinamide adenine dinucleotide (phosphate) [NAD(P)H] oxidases; ragweed SPPs activate human monocyte-derived dendritic cells, providing a direct route from bronchially deliverable particles to antigen-presenting-cell programming [147]. In preclinical systems, pollen-derived NAD(P)H oxidase rapidly generates reactive oxygen species, promotes oxidative stress and neutrophil recruitment, and amplifies antigen-driven inflammation. Removal of oxidase activity attenuated airway inflammation, decreased mucin-containing epithelial cells, and reduced antigen-specific IgE, whereas adding oxidised glutathione or 4-hydroxy-2-nonenal to purified Amb a 1 increased inflammatory-cell recruitment [148]. The contrast between the relatively weak response to purified Amb a 1 and the stronger response produced when oxidative signals are restored is informative: antigen specificity and tissue danger are separable but cooperative components of the response. Oxidative stress is therefore best positioned as an amplifier of allergen-driven inflammation rather than as an alternative initiating mechanism.

Toll-like receptor 4 (TLR4) and its co-receptor myeloid differentiation protein 2 (MD-2) appear to converge on the same epithelial–neutrophilic interface. Ragweed extract induces TLR4-dependent inflammatory signalling, and MD-2 depletion in human bronchial epithelial cells reduces ragweed-induced CXCL8 secretion [143,149,150]. These observations identify a ragweed-characterised route of epithelial activation, although TLR4 and MD-2 signalling itself is not specific to ragweed. Both can increase epithelial danger signalling and innate-cell recruitment upstream of, or alongside, type 2 sensitisation. Protease activity provides a complementary ragweed-associated mechanism. Amb a 11 is a major papain-family cysteine protease allergen, and functional studies demonstrated that its mature proteolytically active form elicited stronger airway inflammation and type 2 responses than forms with lower proteolytic activity [151,152,153]. Thus, Amb a 11 contributes more than antigenic specificity alone: its enzymatic activity can amplify the inflammatory context in which sensitisation occurs. This places these mechanisms in a functional sequence rather than a list, indicating that SPPs can extend allergen delivery into respirable particle fractions; protease activity can provide an additional inflammatory and adjuvant signal; oxidative and pattern-recognition signalling amplify local danger signals; and dendritic-cell programming connects this perturbed mucosal environment to allergen-specific adaptive immunity. The available studies do not establish a universal in vivo order, but they support convergence at the epithelial–innate interface.

### 7.3. Pollen-Derived Cofactors and Environmental Modulation

Adenosine provides particularly direct evidence that ragweed pollen cannot be reduced mechanistically to Amb a 1 alone. In an experimental ragweed model, whole pollen extract—but neither purified Amb a 1 nor the low-molecular-weight fraction alone-induced the complete inflammatory phenotype, including pulmonary inflammatory-cell infiltration, a type 2-associated cytokine signature and impaired lung function. Enzymatic removal of pollen-derived adenosine attenuated ragweed-induced inflammation, while human neutrophils and eosinophils migrated toward supernatants from ragweed-stimulated bronchial epithelial cells only when adenosine was present [154]. Pollen-associated lipid mediators provide an additional, although less ragweed-specific, level of modulation by influencing dendritic-cell programming, granulocyte activity and type 2 polarisation [154,155,156]. These cofactors are therefore most plausibly interpreted as modifiers of immune programming and cell trafficking rather than stand-alone drivers of ragweed allergy. Their contribution is likely conditional on the simultaneous presence of allergen, epithelial perturbation, and a susceptible host, which may help explain why whole pollen or pollen extract can produce responses not reproduced by a purified major allergen alone [154].

Environmental conditions can alter several of these upstream processes before inhalation occurs. Experimental ozone exposure significantly increased NAD(P)H oxidase activity in *A. artemisiifolia* pollen without increasing Amb a 1 expression. In contrast, pollen produced by ragweed grown under elevated CO_2_ has elicited more severe allergic lung inflammation than pollen produced under ambient CO_2_ conditions [81,157]. Growth conditions can also change allergen composition and potency, linking the plant environment to the biochemical quality of the inhaled material. At the molecular level, naturally occurring Amb a 1 isoforms differ in immunogenic and sensitising properties [158], adding another source of variation between nominally similar exposures. This implies that environmental change can modify the biological context in which a given pollen count is experienced through variation in oxidase activity, allergen composition, or co-delivered mediators—without establishing that any single environmental pathway dominates clinical disease.

### 7.4. Shared and Ragweed-Characterised Mechanisms in Comparison with Birch and Grass Pollen

The mechanistic distinction between ragweed, birch, and grass-pollen asthma should not be overstated. All three converge on broadly shared IgE-dependent type 2 effector pathways; the more informative differences lie upstream, in the molecular composition and physicochemical context of the inhaled pollen. Molecular diagnosis supports distinct source-marker repertoires represented by Bet v 1 for birch, Phl p 1 for timothy grass and Amb a 1 for ragweed [159]. Birch sensitisation is strongly centred on Bet v 1, a major PR-10-family allergen that can interact with and traverse airway epithelial model systems [160]. In contrast, grass-pollen sensitisation commonly involves group 1 allergens such as Phl p 1, members of the β-expansin family [161]. Ragweed presents a different molecular repertoire: recent patient-level data confirm the predominance of Amb a 1-specific IgE in genuine ragweed allergy [106]. At the same time, Amb a 11 constitutes a second major allergen with clinically relevant IgE reactivity [102]. Amb a 11 is a papain-family cysteine protease, and experimental comparison of its active and protease-inhibited forms showed that enzymatic activity materially increases airway inflammation and type 2 allergenicity [103,151]. The clinical relevance of this component is supported by a recent cohort of 150 ragweed-allergic patients, in whom Amb a 11 sensitisation was detected in 68.7% and was associated with more severe asthma symptoms (OR 4.71, 95% CI 1.81–12.21) [102]. This association does not establish protease activity as the direct cause of asthma severity, but it strengthens the clinical importance of a ragweed major allergen with enzymatic activity. Other direct comparative experiments also indicate that these upstream pollen properties differ quantitatively rather than constituting strictly taxon-specific pathways. Under the same osmotic-shock conditions, ragweed SPPs retained strong IgE-inhibitory activity against Amb a 1 (81.2%), whereas grass SPPs showed more variable allergen retention, including 38.3% inhibition for Phl p 1; birch SPPs did not inhibit Bet v 1-specific IgE in that assay [162]. These values reflect an experimental SPP preparation rather than clinical potency, but they demonstrate that allergen retention within experimentally generated subpollen particles differs among pollen taxa [162].

Ragweed pollen also provides a well-characterised example of allergen delivery together with intrinsic inflammatory cofactors. Upon hydration, ragweed pollen releases SPPs containing allergenic proteins, including Amb a 1, and functional NAD(P)H oxidase activity; these particles can activate human monocyte-derived dendritic cells [147]. Similarly, NAD(P)H oxidase activity is not unique to ragweed. In a cross-species experimental comparison, oxidase activity was detected in ragweed, grasses and birch but differed in magnitude and localisation, with the tested samples ranking ragweed > grasses > birch, respectively [163].

Taken together, current evidence does not support a unique downstream “ragweed asthma pathway”. Ragweed-, birch- and grass-pollen asthma converge on shared IgE-dependent type 2 immunity. Their mechanistic distinctions arise principally upstream: the dominant molecular allergens differ; Amb a 11 adds a clinically relevant cysteine-protease component to the ragweed repertoire; allergen retention within respirable subpollen material differs among pollen sources; and pollen oxidase activity varies quantitatively and spatially between taxa. In ragweed, these properties converge with experimentally demonstrated TLR4 and MD-2-associated neutrophilic signalling and pollen-derived adenosine to shape the airway environment in which allergen-specific type 2 immunity develops. Importantly, several of these mechanisms are not exclusive to ragweed; rather, they are particularly well-characterised in this pollen system and collectively define the biological context of ragweed exposure.

Within this comparative synthesis, the evidential strength of individual ragweed-associated mechanisms is unequal. The available data support a phase-dependent hierarchy in which allergen-specific recognition provides the immunological specificity of disease, whereas particle characteristics, epithelial injury and pollen-derived cofactors determine the efficiency and inflammatory context in which that specificity is expressed. The IgE–FcεRI/type 2 effector axis has the strongest direct support in human disease, including controlled airway-challenge studies demonstrating local IL13 expression and IL-5-associated eosinophil recruitment [123,124]. By contrast, several proposed upstream mechanisms are supported predominantly by human-cell systems, ex vivo observations or experimental models. Ragweed SPPs activate human dendritic cells, MD-2-dependent signalling has been demonstrated in human bronchial epithelial cells [150], and pollen-derived adenosine influences human granulocyte migration [154]; however, the quantitative contribution of these pathways to naturally occurring human asthma remains unresolved. Experimental manipulation of pollen NAD(P)H oxidase, protease activity and other pollen-associated cofactors provides stronger evidence for biological causality within model systems [143,148,149,150,151,152,153], but not yet for their relative importance in patients.

These mechanisms are most coherently interpreted as a sequential and convergent network. At the exposure interface, particle size determines where allergenic material can be deposited: intact pollen predominantly affects the upper airway, whereas respirable subpollen fractions can extend allergen delivery into the lower respiratory tract [147]. At the epithelial interface, Amb a11 protease activity can amplify the inflammatory context of allergen exposure, while NAD(P)H oxidase-derived reactive oxygen species and TLR4/MD-2 signalling promote cellular stress, chemokine production, and neutrophil recruitment [143,148,149,150,151,152,153]. These events are mechanistically distinct but converge functionally by increasing allergen accessibility and shaping the local milieu in which dendritic cells encounter pollen antigens. Adenosine and pollen-associated lipid mediators act at a different level: rather than providing antigen specificity, they can modify dendritic-cell programming, granulocyte trafficking and immune polarisation [154,155,156]. Epithelial alarmins therefore constitute a plausible point of convergence between physical injury, innate recognition and subsequent type 2 immunity. Once sensitisation is established, however, allergen-mediated cross-linking of FcεRI-bound IgE on mast cells and basophils becomes the principal mechanism of immediate elicitation, with IL-5-associated eosinophilia, ILC2 activity, epithelial alarmins and neuroimmune feedback contributing predominantly to amplification and persistence of the late-phase response [123,124,131,134,141,142].

This temporal distinction is important when interpreting experimental studies. An intervention that suppresses sensitisation by removing an oxidative or proteolytic co-signal does not demonstrate that the same pathway dominates acute symptoms in an already sensitised patient. Conversely, effective IgE- or type 2-directed treatment does not establish that epithelial and innate events are irrelevant, because those events may determine the amount, anatomical distribution and inflammatory context of allergen encountered before FcεRI-dependent activation occurs. Thus, relative contribution is likely to be state-dependent rather than fixed: particle delivery, barrier integrity and innate co-signalling may exert greater influence during sensitisation and exposure amplification, whereas IgE–FcεRI signalling dominates immediate re-exposure and type 2 cellular circuits sustain subsequent inflammation.

A further consequence of this model is that neither pollen counts nor molecular sensitisation profiles alone should be expected to predict disease intensity. Two individuals with similar Amb a 1-specific IgE profiles may receive biologically different exposures if airborne allergen content, particle-size distribution, protease or oxidase activity, and co-pollutant conditions differ; conversely, similar atmospheric exposure may produce divergent clinical responses according to sensitisation pattern, epithelial susceptibility, and pre-existing airway disease. This provides a mechanistic explanation for the observed decoupling between intact pollen concentration, airborne allergen load, and clinical response discussed earlier in this review.

## 8. Limitations and Unresolved Questions

Several limitations constrain the interpretation and clinical translation of the proposed framework. Most importantly, evidential directness remains uneven: the allergen-specific IgE/type 2 axis is supported by human challenge and clinical evidence, whereas the quantitative contribution of oxidative, TLR4/MD-2, protease, metabolic, and neuroimmune pathways relies predominantly on experimental systems. Their effect sizes, temporal ordering, and interactions in the human lower airway therefore remain unresolved, and the proposed hierarchy should not be interpreted as a quantitatively validated causal model.

Second, exposure assessment remains incomplete. Standard Hirst-type pollen monitoring is indispensable for surveillance and forecasting, but pollen counts do not resolve airborne Amb a 1, pollen allergen potency, respirable allergen-bearing fractions, atmospheric processing, or indoor–outdoor exposure differences. Conversely, molecular allergen measurements without concurrent pollen, meteorological, pollution, and particle-size information cannot characterize the full inhaled exposure. The key limitation is therefore not the use of standard pollen monitoring itself, but the absence of routinely integrated exposure layers.

Third, component-resolved sensitisation profiles are not yet routinely linked to longitudinal clinical outcomes. Prospective validation should combine molecular diagnostics with symptom and medication scores, rhinitis and asthma severity, lung function, exacerbations, and treatment response, while simultaneously measuring pollen concentration, airborne allergens, pollen allergen potency, meteorology, pollution, and particle-size-resolved allergen-bearing fractions.

Last but not least, geographical replication will also be important because the correspondence between molecular sensitisation, atmospheric allergen load, and clinical outcomes may differ between long-established high-exposure regions and newer or lower-exposure invasion fronts. Validation should therefore include cohorts spanning contrasting exposure histories rather than relying on a single hotspot population.

## 9. Conclusions

Common ragweed risk is generated by a chain of ecological, atmospheric, molecular, and host processes rather than by plant occurrence or pollen concentration alone. Invasion history, disturbance, seed-bank persistence, and adaptive shifts in flowering determine where and when pollen is produced, whereas atmospheric transport, allergen potency, and respirable fractions determine the biologically effective inhaled dose. Finally, four conclusions emerged from the available evidence. First, future spread remains conditional on the interaction of repeated introductions, propagule pressure, disturbed habitats, adaptive genetic variation and persistent seed banks, which together support establishment, persistence and further range expansion. Second, clinically relevant exposure is not solely dependent on pollen abundance, but on pollen-season timing, pollen transport, the presence of allergen Amb a 1 in the pollen, the level of allergen, the allergen-bearing size fraction, meteorology and pollution. Third, molecular allergen profiling is important for distinguishing genuine ragweed sensitisation from cross-reactive IgE responses. Amb a 1 remains the principal marker of genuine ragweed sensitization, whereas Amb a 11, defensin-like allergens, panallergens, and carbohydrate-related IgE signals require context-specific interpretation. Fourth, ragweed-related airway disease reflects the interaction of IgE-dependent type 2 immunity with epithelial danger signals, oxidative stress, protease activity, innate immune activation, and environmentally modified allergen delivery.

Practical implications include the need to monitor and manage ragweed as an invasion–exposure–disease system. Surveillance should connect plant distribution, phenology, pollen counts, levels of airborne allergens, molecular sensitization profiles, symptoms, lung function, asthma outcomes and control interventions. This integration would enhance the ability to predict, improve source attribution, identify high-risk invasion fronts and strongly aid in prevention strategies aimed at reducing plant establishment and aeroallergen exposure relevant to human health.

## Figures and Tables

**Table 1:** Ragweed allergen molecules with mechanistic and clinical takeaways.

Allergen	Molecular Identity/Family	IgE Reactivity Reported	Key Takeaways	Approx. MW	References
**Amb a 1 and Amb a 1.01**	Pectate lyase family; major pollen allergen	>90%	Best single marker of genuine ragweed IgE; multiple isoforms with varying IgE reactivity	~38 kDa	[21,105]
**Amb a 3**	Reported as plastocyanin (minor allergen)	30–50%	Adds endotype structure; not a primary attribution marker.	~11 kDa	[114]
**Amb a 4**	Defensin-like protein linked to a polyproline-rich region; clinically important in weed pollen-food syndromes (minor allergen)	20–40%	Homologous/cross-reactive with Art v 1; useful in mugwort versus ragweed attribution	~28–30 kDa	[104]
**Amb a 5**	Group 5 minor allergen	10–15%	Low-frequency breadth contributor	~5 kDa	[21]
**Amb a 6**	Type I Non-specific lipid transfer protein (minor allergen)	~20–35%	Noted as distinct among nsLTPs with limited cross-reactivity	~10 kDa	[21,100]
**Amb a 7**	Reported as plastocyanin (minor allergen)	15–20%	Adds endotype structure; not a primary attribution marker	~12 kDa	[115]
**Amb a 8**	Profilin (minor panallergen)	20–35%	Strong cross-sensitization potential	~14 kDa	[116]
**Amb a 9**	Polcalcin-like calcium-binding protein (minor panallergen)	10–15% (assay/cohort-dependent)	Cross-source homology and potential attribution confounding	~9 kDa	[117]
**Amb a 10**		10–30%		~17–18 kDa	[115,118]
**Amb a 11**	Cysteine protease (major allergen)	~70%	Associated with more severe asthma in the cited material; protease biology invites barrier-relevant hypotheses	~37 kDa mature; zymogen ~52 kDa	[102]
**Amb a 12**	Enolase (minor allergen)	≈41–68%	Best framed as a candidate pan-allergen with variable clinical weight across assays/cohorts	~48 kDa	[87]

Reported IgE reactivity ranges are compiled from cohort-based studies where available and otherwise from review syntheses; ranges should be interpreted as assay- and cohort-dependent rather than as population prevalence. Abbreviations: Amb a, *A. artemisiifolia* allergen; Art v, *Artemisia vulgaris* allergen; IgE, immunoglobulin E; MW, molecular weight; kDa, kilodalton; nsLTP, non-specific lipid transfer protein.

## Data Availability

The data presented in this study are not readily available as it is part of an ongoing doctoral thesis. Requests to access the data should be directed to the corresponding author.
